# HALO: Study protocol for a single-case experimental design study evaluating the moderating impact of a befriending intervention on the association between loneliness and health in older adults

**DOI:** 10.12688/hrbopenres.13104.1

**Published:** 2020-09-04

**Authors:** Caoimhe Hannigan, Paul Hanly, Frank Kee, Brian Lawlor, Eimile Holton, Cathal Walsh, Thomas Scharf, Robert Coen, Vicky Leatham, Sean Moynihan, Keith Lane, Joanna McHugh Power

**Affiliations:** 1 School of Business, National College of Ireland, Mayor St, Dublin 1, Ireland; 2 Centre for Public Health, Queen's University Belfast, Belfast, BT12 6BJ, UK; 3 School of Medicine, Trinity College Dublin, University of Dublin, Dublin 2, Ireland; 4 Department of Maths and Statistics, University of Limerick, Limerick, Ireland; 5 Population Health Sciences Institute, Newcastle University, Newcastle, UK; 6 St James's Hospital, Dublin 8, Ireland; 7 ALONE, Pleasants Street, Dublin 8, Ireland; 8 Department of Psychology, Maynooth University, Kildare, Ireland

**Keywords:** Loneliness, mixed methods, befriending, social support, older adults, single-case experimental design, generalised additive models, grounded theory

## Abstract

**Background**: Loneliness in later life is often addressed with befriending interventions, yet evidence for their effectiveness is limited. Meanwhile it is known that loneliness has a deleterious impact on health. The aim of the study is to evaluate whether a befriending service for older adults mitigates the impact of loneliness on health outcomes, and to identify mechanisms through which befriending interventions might impact upon health.

**Methods**: A mixed methods design is used. The quantitative component utilises an AB single-case experimental design, to gather intensive longitudinal data. These data will be analysed using a generalised additive modelling approach. The qualitative component of the study uses semi-structured dyadic interviews, structured and analysed according to the principles of constructivist grounded theory. Findings will then be triangulated according to an existing mixed methods integration protocol.

**Discussion**: This mixed methods design has the potential to inform national and international policy in relation to befriending interventions for older adults. In addition, there is the potential for study results to inform our theoretical understanding of the nature of the relationship between loneliness and health.

**Trial registration:** ClinicalTrials.gov identifier NCT04301167 (10 ^th^ March 2020). Protocol version 1.1, 26 ^th^ June 2020.

## Introduction

Befriending services are often used to provide social connection to individuals who are socially isolated. The service typically involves trained volunteers being matched with a service user in a structured manner, e.g. for weekly visits. Befriending services may improve health, since elective relationships have a positive impact on health ( Golden *et al.*, 2009). Befriending services have also been shown to reduce loneliness ( Lawlor *et al.*, 2014). Befriending may also benefit brain health, since social relationships are associated with a reduced risk of developing dementia ( Kuiper *et al.*, 2015; Morley *et al.*, 2015).

However, the theoretical basis for how befriending might impact health is poorly understood ( Lester *et al.*, 2012). The causal cascade model has been used to explain the impact of social engagement on health ( Berkman *et al.*, 2000) and cognitive decline ( Zunzunegui *et al.*, 2003) and could constitute a model of the relationship between befriending and brain health. In this model, social engagement confers a health benefit to individuals at a series of levels from the macro (cultural norms, urbanisation), the mezzo (social network size, reciprocity of ties) to the micro (emotional social support, intimate contact), which all influence health or cognitive functioning via behavioural (e.g. help-seeking behaviour, diet), psychological (e.g. self-esteem), and physiological (e.g. immune system function, allostatic load) pathways.

Befriending may improve health partly via its effect on loneliness ( McGoldrick *et al.*, 2017), which affects 10% of older Irish adults ( Harvey & Walsh, 2016). Reducing loneliness is a desirable outcome because of its associations with various undesirable outcomes including depression ( O'Luanaigh & Lawlor, 2008), dementia ( Wilson *et al.*, 2007) and poor quality of life ( Ekwall *et al.*, 2005). Researchers have predicted that befriending services could reduce dementia by 0.21% through reducing loneliness ( Owen *et al.*, 2016). However, this prediction has not been formally evaluated.

As has been highlighted previously ( Harvey & Walsh, 2016), loneliness has mostly been ignored in Irish health policies, and services that address loneliness are not delivered in a centralised or coordinated fashion. This situation differs to the UK, where a befriending sector is established, alongside a national campaign to end loneliness, which *inter alia* promotes befriending to decrease loneliness. Members of the research team have previously designed and evaluated two psychosocial interventions, one of which found that befriending alleviated loneliness ( Lawlor *et al.*, 2014). We also evaluated an existing befriending service and found that it was perceived as an effective way to reduce loneliness ( Hannigan *et al.*, 2015).

The proposed research is timely since it responds to recommendations made in the Institute of Public Health’s recent report on loneliness in Ireland ( Harvey & Walsh, 2016), and to an evaluation finding that Ireland has poor availability of services to reduce loneliness ( Burke, 2015) as well as recent efforts to martial government funding towards loneliness services ( Loneliness Taskforce). The research will add to the scientific evidence base for use of befriending interventions to improve health, and will help elucidate the mechanisms through which befriending improves health. Existing theories provide a clear scientific rationale for hypothesising that befriending will impact health, and may moderate the impact of loneliness on health.

### Aims and objectives

**Overall study aim:** To improve understanding of the health-related impacts of befriending on older adults, and to understand the role of loneliness in this regard.

**Hypothesis 1:** Befriending services have a positive impact on health-related quality of life and brain health.

**Hypothesis 2:** Receipt of a befriending service moderates the negative impact of loneliness on health-related quality of life and brain health.

**Objective of qualitative component:** To explore potential mechanisms by which befriending impacts health in a series of dyadic and individual semi-structured interviews with recipients of a befriending service and their partnered befrienders.

## Methods

This study was registered retrospectively on ClinicalTrials.gov (identifier NCT04301167) on 10 ^th^ March 2020. As per ClinicalTrials.gov guidelines, registration can be completed at any point during the project lifespan except in the case of medical trials. In the case of the current study, registration was delayed because of staffing issues.

### Design and study setting

The study uses a mixed methods design. Quantitative methods are used to investigate whether befriending moderates the impact of loneliness on health-related quality of life and brain health. Qualitative methods are used in an exploratory supplementary fashion to explore potential mechanisms through which befriending impacts health. A mixed methods analysis will triangulate and integrate findings across the quantitative and qualitative approaches to arrive at an empirically justifiable theory of the mechanisms through which befriending might impact health. For the quantitative study component, participants can choose to have their initial informed consent meeting in their homes or in testing suites in the host research institution. Participants are eligible if they live within an hour’s drive of Dublin, Ireland. Thereafter, data collection is phone-based. For the qualitative study component, interviews can be conducted in person or over the phone, depending on participant preferences.

### Sample

Quantitative component: Sample size was calculated to ensure that the study was sufficiently powered. Power in single-case experimental designs is a function of the following: sample size (number of observations per phase multiplied by number of participants), intraclass correlation (ratio of between-case variance to total, or within and between case variance), auto-correlation, effect size to be detected, and the nominal Type 1 error rate ( Shadish *et al.*, 2014a). A rigorous approach described by Shadish and colleagues (2014a) to calculating sample size uses the alternative d-statistic to the traditional statistic used in between-subjects designs for single case designs. Our primary outcome of interest is health-related quality of life, which will be measured using the EQ-5D scale (see Outcomes). The minimally important difference in this scale has previously been calculated as 0.074 ( Walters & Brazier, 2005) and norms of the EQ-5D have yielded a standard deviation of 0.23 ( Kind *et al.*, 1999). These figures were used to estimate an effect size (using Shadish’s alternative d-statistic) of 0.32. Using this figure in a simulation macro provided by Shadish and colleagues (2014a), with intraclass correlation set to a conservative estimate of 0.5, autocorrelation set to same, and alpha set to 0.05 for a multiple baseline design) with 13 observations (i.e. a period of six months) per participant, and desired power of 0.8, a sample size of 85 is required. This sample size also provides sufficient power (0.9) for the secondary outcome, calculated using the same approach described above.

Qualitative component: 10–15 befriendee-befriender pairs will be recruited via the collaborating organisation. Sample size estimation in constructivist grounded theory is guided by data review after 3–4 interviews are conducted to assess thematic saturation ( Charmaz, 2006).

### Sample selection and recruitment

Quantitative component: Participant recruitment is purposive and non-probability. Participants are recruited via ALONE, a charity organisation based in Dublin who offer, among other services, a befriending service for older adults. Participants will be recruited from the pool of individuals who contact ALONE to enquire about these befriending services. Typically, such individuals are referred from other services (e.g. GP services) and a visit is arranged to discuss service uptake. For the period of the proposed study data collection, ALONE staff members will include two additional questions to their routine visits – whether individuals self-report as lonely, and whether they would be interested in participating in an evaluation of the services. If individuals wish to be involved, they will receive information followed by a phone call from the project research assistant. If the participant is screened as eligible for the study, and is willing to participate, an initial data collection appointment will be made (with fortnightly appointments to be scheduled following this point for the remainder of the participant’s involvement in the study, up to a total of 11 post-intervention data collection points, or a period of six months).

An average of 35 (range 18–66) individuals were referred to ALONE each month between May 2016 and April 2017 (with a positive trend in numbers, likely owing to increased recruitment efforts on the part of ALONE). Of these, a conservative estimate is that 50% (17) would be interested in receiving a befriending service. A recent evaluation of ALONE’s befriending service found that 40% of service users defined themselves as lonely prior to service uptake ( Burke, 2015). As such, 10 participants can be expected to self-identify as lonely, and thus be considered to be eligible for further screening into the study. Assuming a response rate of 50%, approximately five individuals could be recruited per month, meaning that the target of 85 participants is likely to be reached across 17 months of data collection. Given that six months are required as the follow-up period, this means that a minimum of 23 months’ data collection period is required.

Qualitative component: Participant recruitment is purposive, non-probability sampling, from those already using the befriending service.

Inclusion criteria for quantitative component:

- Living in the greater Dublin area.
- Participants must have contacted ALONE with an interest in receiving befriending services, and must be on a waiting list to receive such services at the time of their trial registration.
- Participants must be aged 60 and over.
- Participants must have the ability to provide informed consent.
- Participants must self-report that they experience loneliness “at least sometimes”.

Exclusion criteria for quantitative component:

- Participants must not self-report receipt of a diagnosis from a medical doctor of any of the following: intellectual disability including autism spectrum disorder or psychotic disorder, dementia, serious memory impairment
- Participants must not live further than an hour’s drive from Dublin.

Inclusion criteria for qualitative component:

- Participants to be recruited in pairs must have been paired as befriender-befriendee for at least six months, and the befriendee must have been in receipt of any befriending from the service for a minimum of one year.
- Living in the greater Dublin area.
- Befriendees must be aged 60 or over.
- Both pair members must have the ability to provide informed consent.
- Both pair members must have sufficient language ability to engage in the interview.

### Intervention

The intervention is a befriending service. This service provides companionship to older people who would like extra social contact, through a weekly visit from a volunteer befriender. The volunteer befriender provides companionship and small practical supports where necessary and appropriate. The timing of the weekly visit, along with the nature of these visits (e.g. activities the befriender-befriendee pair may wish to complete together), is agreed between the older person and their volunteer befriender. ALONE recruit volunteers to provide the befriending service. Recruitment, training, and perpetual support is offered by staff at ALONE whose remit is to manage the befriending service. ALONE also have staff members who discuss a range of potential services and benefits available in the community and through ALONE with the older adults who contact them. At this initial meeting, older adults are told about the befriending service and can opt in to receiving this service. If an older adult expresses interest in the befriending service, they are then told about the research study running concurrently with the service. They are told that the service is available to them regardless of whether they are interested in the research study, and that their involvement in the study has no bearing on their receipt of the service. If the older adults are interested at this point in the research study, ALONE staff request verbal consent to pass their contact details to the research team. ALONE befriending service staff contact the older adults to discuss the befriending service with them at this point. They are made aware that the goal is for service users to be matched to a befriender as soon as possible and that before this match takes place at least two observations will be made (one initial, and one after a period of two weeks, and thereafter). If a participant is matched before two observations are delivered, this means that the participant has exited the baseline period of the trial early and their data may be excluded from the final dataset. Participants are asked about any preferences they may have in relation to their befriender by ALONE staff, who then attempt to find a match for them. The befriending match also takes into account location of the service user and potential befriender. Once a match has been made, the service user is informed, and staff from ALONE accompany the befriender to the home of the service user for an initial visit. Adverse events arising from participants’ receipt of the befriending service will be the responsibility of ALONE, as is normal practice.

Receipt of the befriending service intervention is at the discretion of the service user. They are permitted to withdraw from the service at any time if they wish, or request an alternative befriending partner. Should participants withdraw from the befriending service they will be invited to continue with data collection since this will facilitate the examination of N-of-1 ABA (no intervention, intervention, no intervention) phased analysis of the trajectories of outcomes. After completion of the study data collection, it is anticipated that participants will continue to receive the intervention indefinitely, at their discretion.

For the purpose of this study, participants are their own comparators. The reason for this is that the service under investigation is already running in the community and desirable to potential participants. As such it would be unethical to create a control arm as a comparator since these participants would then fail to receive the befriending service that they have requested in good time. The study design permits the research team to measure study outcomes before and after the intervention (befriending service) commences delivery, for a six-month period. As a result, participant outcomes after commencement of the intervention can be compared to those outcomes before this event.

### Adherence

In relation to involvement in the research study, the research assistants will make every effort to be flexible with their appointments for observations, which take place every two weeks. If participants miss observations, the research assistants will provide opportunities for additional observations at the end of the expected 26 weeks, and if this is not possible, and the participant is overly taxed with their engagement in the study, research assistants will suggest that they participate at least in the final time point (after 26 weeks) since our statistical modelling techniques can still garner trends based on these incomplete data. 

No prohibitions are placed on study participants or their befrienders during the trial. Since participants are linked in with general support coordinators over the course of their engagement with the trial, they may be in receipt of other services as requested/required. Support coordinators in ALONE link older adults with a range of nationally available services. The trial ends six months after registration for participants. However, the service persists for as long as participants require or desire. ALONE recommend a year-long commitment in the first instance to befrienders, so in most cases participants will retain their befriending service visits from the same volunteer. However, it is possible that befrienders will withdraw or that participants will request a change in befriender during the trial lifespan. In this event, data collection will continue, and the withdrawal or change in befriender will be modelled as a covariate in the statistical analysis.

### Outcomes

***Primary outcome: Changes in health-related quality of life over six months.*** EuroQOL (quality of life) five-dimension scale with five response levels (EQ5D-5L) ( Rabin *et al.*, 2014). This scale is a well-validated scale of health-related quality of life, measuring five dimensions of health: mobility, self-care, usual activities, pain, and anxiety/depression. The score is suitable for repeated measures and yields scores of five levels of severity for each dimension. Scores can then be combined into a single summary index of utility. A score of 0 on the EQ5D indicates death, while a score of 1 indicates perfect health. The EQ5D-5L is administered at each of 13 observations made two weeks apart for a minimum of two observations before the intervention begins and a maximum of 11 observations after.

***Secondary outcome: Changes in brain health over six months.*** Repeated testing of cognitive functioning can result in practice effects, and two strategies are put in place to minimise these. First, specific analytic strategies will be used to detect and account for practice effects in the data. Second, tasks are chosen to minimise the effects of practice on outcomes ( Goldberg *et al.*, 2015). Two alternating versions of the verbal fluency task from the Delis Kaplan Executive Functioning System ( Delis *et al.*, 2001) are available for this purpose, and will be used alternately at observations across 13 timepoints.

***Time-varying moderator: Change in loneliness over six months (13 observations).*** Loneliness is measured using the modified five item University of California Los Angeles (UCLA) loneliness scale ( Russell, 1996) at each of 13 observations made two weeks apart for a minimum of two observations before the intervention begins and a maximum of 11 observations after. In this scale, five items ask participants to rate how frequently they experience each aspect of loneliness, and scores are summed to yield a range of 0-10 (with higher scores indicating more loneliness). The scale is well-validated with high internal consistency (alpha = 0.89) and test-retest reliability (r = 0.73 over two months), and has been used in cohort studies of ageing internationally including the Irish Longitudinal Study on Ageing ( Kenny *et al.*, 2010).

***Time-varying covariate: Change in social isolation.*** The revised Lubben Social Network Scale is a short (six item) and well-validated scale to measure social isolation in older adults ( Lubben & Gironda, 2004). Six questions probe the level of received support from family and friends. Responses are equally weighted and give scores of 0-30 with higher scores indicating more perceived social support/less isolation.

For all above outcomes, change over time is the analysis metric. All outcomes are measured a maximum of 13 times from which trends can be created. Trends will be treated as random effects to account for inter-individual variation and intra-individual variation. All outcomes were chosen with clinical relevance in mind. Befriending services are typically provided because of loneliness so it is crucial to explore whether loneliness typically reduces as a result of uptake in the service. Health-related quality of life and brain health are two of the most critical outcomes for older adults.

### Procedure

***Obtaining consent.*** The trial research assistants will be trained by the trial Principal Investigator to obtain informed consent. The research assistant will obtain informed consent at the first observation point, which will normally take place in the home of the participant. After participants express an interest in the study to the ALONE service, the research assistant will provide them with information leaflets and an informed consent form by post (see *Extended data*; Hannigan *et al.*, 2020). Following a period of at least seven days after the information is likely to have been received by the participant, the research assistant will contact the potential participant and at this point, if they are still interested in the study, conduct a short screen (based on the above inclusion and exclusion criteria) by phone. If eligible, the potential participant will then receive a home visit from the research assistant to discuss informed consent and if consenting, to conduct the first observation. Once the research assistant is satisfied that the potential participant has read (or had read to them) and fully understood the information leaflet, they will proceed to obtain written informed consent. The written consent informs participants that they are permitted to withdraw from the study at any time, and they will take their own copy of this consent form and information leaflet, signed by themselves and by the research assistant, from this first meeting. Contact details of the broader research team are available on this leaflet should participants wish to discuss the study or their involvement further.

**Figure 1.**
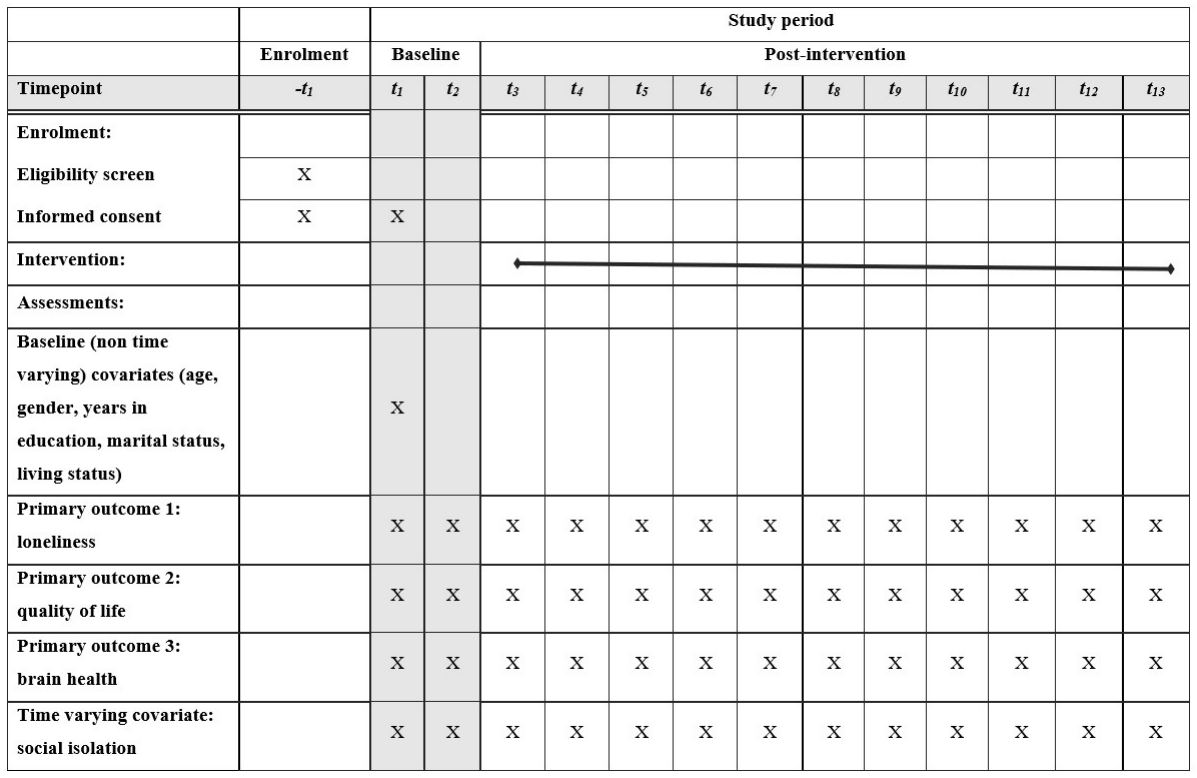
Schedule of enrolment, interventions, and assessments for HALO study participants.

***Participant timeline.*** The participant timeline is illustrated in the schematic diagram (see Figure 1). Participants can be enrolled to the study between November 2018 and January 2021. Enrolment involves a washout period of seven days after provision of information leaflets, to allow participants time to consider their potential participation. Enrolment follows a positive eligibility screen, and informed consent is taken at the point of enrolment (t _1_). The first observation is made at this time point too. This observation includes baseline (non-time varying) covariates. These covariates are age, gender, number of years in education, marital status (responses are single, divorced, separated, married living with spouse, cohabiting with partner, divorced), living status (living alone, living with spouse only, living with child/stepchild/grandchild, living with another relative, living with unrelated individuals). The outcomes and time-varying covariate (social support) listed above are measured at each of the 13 observations. The first observation is made in person while all subsequent observations are made by phone.

***Data collection and management.*** Data for the quantitative aspect of the study will be collected at 13 time-points, using a structured assessment form. The baseline assessment is administered during an initial visit to the participant’s home, where informed consent will first be obtained. All subsequent assessments take place by phone, at fortnightly intervals over a period of six months. The measures included in the quantitative assessments are described in detail in the outcomes section above. The project research assistant will collect and manage all quantitative assessment data. A number of measures are in place to promote data quality. Written standard operating procedures are used for data collection and input, including scripts and instructions for administration of all measures. The research assistant is experienced in conducting psychosocial and neuropsychological assessments with older adults, and also received initial training from the senior investigators to ensure competence in administering the specific study measures.

Qualitative data will be collected during a semi-structured dyadic interview with befriendee-befriender pairs (interview guides are available as *Extended data*; Hannigan *et al.*, 2020). These interviews will be conducted by the project research assistants and senior investigators, who are experienced in collecting qualitative interview data. All interviews will be audio recorded using a digital recording device.

Since data collection takes place every two weeks, the research assistant will be in regular telephone contact with research participants. To promote retention, phone assessments will be scheduled for a regular time/day that the participant has identified as being convenient. In the case of a missed observation, the research assistant will make multiple follow-up contacts in an attempt to complete the assessment. The statistical modelling techniques being used can accommodate incomplete data, therefore continued engagement will be encouraged even if an observation is missed. If a participant is overly taxed with their engagement in the study, research assistants will suggest that they participate at least in the final time point (after 26 weeks).

Quantitative data is initially collected using written assessment booklets. Data from these booklets are then entered into an electronic file for further processing and analysis. Quantitative data entry is conducted by the project research assistant. Data entry and initial processing is conducted using Microsoft Excel; an Excel-based data entry system was developed that includes automated processes designed to reduce human error during data entry and processing. These include instructive prompts, automated range checks for all values and automated calculation of total/scale scores etc. Ongoing data audit procedures are also used by research team members not including the project PI to ensure data quality, including cross-checking of scoring and data entry by a second member of the study team for a random sample (10%) of cases.

Qualitative interview data is recorded using a digital recording device. All audio recordings will be transferred to a secure PC system immediately after interviews and then deleted from the digital recording device. These audio recordings will then be transcribed verbatim for analysis. Transcripts will be checked against audio recordings to ensure data quality.

All research data in both written and electronic records are stored under a unique participant ID code, and no identifying information is included in either written or electronic data files. Written assessment booklets are stored securely in a locked filing cabinet within a locked office at the host institution. All electronic data files (quantitative data file, qualitative audio recordings and transcripts) are password-encrypted and stored on a password-protected, secure PC system within locked offices at the host institution. Access to these files is limited to members of the research team. All data management procedures have been approved by the relevant Research Ethics Committee within the host institution. A Data Protection Impact Assessment (DPIA) was also submitted and approved by the Data Protection Office within the host institution. Should any protocol amendments be required throughout the duration of the study, the PI will take responsibility to inform the research ethics committee (and apply for ethical approval afresh, if required), the funder, ClinicalTrials.gov, journals, collaborators, and trial participants if necessary. The named collaborators (authors of this protocol) will have access to the final dataset.

***Confidentiality.*** To maintain confidentiality, all participants are assigned a unique study ID code. All study data is collected and stored under this ID code, separate from personal identifying information. ALONE will provide the research team with contact details (name and phone number) for potential participants, subject to the consent of the participant for that information sharing. At this point a study ID code will be assigned to the potential participant. The research assistant will maintain a ‘key’ file which will link the participant’s study ID code to their name and personal contact information. Further contact information such as addresses for home visits will be collected by the research assistant at study enrolment, and added to this ‘key’ file. This electronic, password-encrypted file will be stored on a password-encrypted, secure PC system. Access to this file will be limited to the research team, and will be used only to arrange home visits, or to allow the research team to withdraw data from the study if requested. Participants provide informed consent for the processing and storage of their personal data as outlined above.

### Analytic plan

In order to test hypothesis 1, that befriending services will lead to a positive change in health-related quality of life and brain health, two separate generalised additive models will be performed. Data are longitudinal, with 13 time points at which they are collected, before and after a befriending match has been made. The data analysis protocol for the quantitative component of the study is as follows: first, after data collection and audit, visual inspection of plotted data at the individual and group level will be done for both outcomes of interest. Next, pre-intervention and post-intervention trends will be inspected, as well as mean shifts, slope changes, and variability across participants. With respect to verbal fluency as an outcome, data will be analysed for practice effects using published guidelines ( Vivot *et al.*, 2016) and if practice effects are detected, this should be specified in the final model. The modelling of these practice effects is itself of interest and can inform future research attempting to conduct repeated cognitive measures. If practice effects are considerable, this could represent a risk for the interpretability of the results, but since the cognitive outcome is a secondary measure in the study design, this risk does not affect the overall study feasibility. Inferential analyses will be conducted using a generalised additive model (GAM) ( Shadish *et al.*, 2014b) based on a multilevel approach where time points are nested within participants ( Shadish *et al.*, 2014b). This approach is semi-parametric, and calculates autocorrelations from extracted residuals. Time will be scaled such that point 0 is the end of the baseline (two time points of data collection will be conducted during baseline). The GAM approach improves on some key limitations of the multilevel approach, since with the latter, the functional form of the trend must be known, whereas in the former, a smoothing function can be used to model the trend in the data. The moderation effect (investigating whether befriending moderates the impact of loneliness on health and cognitive function) is modelled as an interaction term: the product of the two predictors (loneliness and befriending phase, operationalised as 0 indicating pre-intervention, and 1 indicating post-intervention). This modelling approach will be deployed using the mgcv package in R Studio software ( Wood, 2011).

The GAM approach is relatively robust to missing data, and imputation can be incorporated into the analysis. An additional advantage of the GAM approach is that equal numbers of data points per participant is not a requirement.

Statistical code will be published with any publication to arise from the study. Participant level data will not be archived, but we hope to make available a covariance matrix arising from the data, which can be shared upon reasonable email request.

The next step in the analysis plan concerns the qualitative data. The qualitative interview data will be analysed according to principles of constructivist grounded theory ( Charmaz, 2006) in three parts: befriender interviews, befriendee interviews, and dyadic interviews. Themes arising from all three types of interview will be integrated and compared as per existing guidelines ( Eisikovits & Koren, 2010). Finally, a mixed methods triangulation protocol will finally be conducted to integrate the qualitative and quantitative findings ( Tonkin-Crane *et al.*, 2016).

### Oversight and monitoring

The trial is being conducted in only one centre so all coordination of trial activities is managed by the core research team at the host institution. Other project governance structures include a Project Management Board and a Project Steering Group. The Project Management Board is made up of the wider project team and meets once per quarter to review project progress and discuss plans for upcoming activities. A Project Steering Group is made up of client representatives from ALONE, representatives from the Befriending Networks Ireland (BNI) Advisory Panel and members of the wider project team. This Steering Group will meet on multiple occasions throughout the project to provide the research team with additional input and perspectives on the outcomes being studied; to ensure that the research topics, planned methodologies, language and format of study materials etc. are appropriate and acceptable; to assume the role of data monitoring; and to advise on and be involved with dissemination activities such as public workshops.

### Ethics

This study has received ethical approval from the Faculty of Health Sciences REC at Trinity College Dublin (Ethical Approval Reference Number: 180501). Any changes to the trial protocol that require an amendment to the existing ethical approval will be submitted in writing to the relevant REC at the host institution. Any proposed changes to the study protocol will be discussed and approved at a meeting of the Project Management Board. If any significant changes are made to the protocol that will impact on participants’ engagement with the study (e.g. changes to eligibility criteria or outcome measures), participants will be contacted to discuss these and a written description of these changes will be posted to participants where appropriate.

### Dissemination plans

Conference presentations and publications in peer-reviewed journals will be one method of dissemination. These will be done following the data analysis and prior to the process of application and translation of study results. Two publications (one describing quantitative outcomes in a health psychology journal, one describing mixed methods findings in a health and ageing journal), and two conference publications (one national - Irish Gerontological Society, and one international - Gerontological Society of America or EU Geriatric Medicine Society) will likely arise from the study with all collaborators (co-authors of this protocol) invited to participate in authorship following ICMJE guidelines on authorship criteria. Dissemination activities tailored to the needs of the knowledge user organisation will also be conducted, such as presenting the research at knowledge user events, and engaging in public relations exercises with personnel from the knowledge user organisation, to raise awareness of the research and of the available befriending services. Policy recommendations will be diffused across stakeholders, and disseminated to policymakers, and findings will finally be disseminated to community members in the form of a launch event with a lay report provided.

### Study status

Recruitment and data collection for both the quantitative and qualitative components of the study are ongoing. Recruitment to the quantitative component began in November 2018. To date a total of 49 participants have been recruited to the quantitative study. Recruitment to the qualitative component began in November 2019 and a total of seven befriender-befriendee dyads have completed qualitative interviews to date. It is anticipated that recruitment for both components will be completed by December 2020.

## Discussion

This protocol describes the pragmatic, embedded evaluation of an existing service which claims to reduce loneliness and mitigate the impact of loneliness on health outcomes for older adults. Currently we are recruiting during the COVID-19 pandemic in which, nationally, older adults are among those being advised to “cocoon” and remain in the home, potentially leading to increased risks of loneliness and social isolation, and higher country-level demands for services to resolve such issues. Inevitably, recruitment has slowed down because fewer services are available to older adults, but recruitment is beginning to recover (June 2020). All data collection and recruitment procedures are, for the time being, conducted by phone to avoid face-to-face visits yielding increased risk of COVID-19 transmission to older adults. It is hoped that the completion of our study will add to the evidence base for the uptake of befriending services for older adults at risk of, or experiencing, loneliness and social isolation.

## Data availability

### Underlying data

No data are associated with this article.

### Extended data

Open Science Framework: HALO: Loneliness and Health: The moderating role of befriending services https://doi.org/10.17605/OSF.IO/YKZTP ( Hannigan *et al.*, 2020)

This project contains the following extended data:

- HALO Consent Form_Qualitative Study.pdf
- HALO Consent Form_Quantitative Study.pdf
- HALO Participant Information Leaflet_Qualitative Study.pdf
- HALO Participant Information Leaflet_Quantitative Study.pdf
- HALO Qualitative Interview Schedule.docx

### Reporting guidelines

Open Science Framework: SPIRIT checklist for ‘HALO: Single-case experimental design study evaluating the moderating impact of a befriending intervention on the association between loneliness and health in older adults’. https://doi.org/10.17605/OSF.IO/YKZTP ( Hannigan *et al.*, 2020)

Data are available under the terms of the Creative Commons Zero “No rights reserved” data waiver (CCO 1.0 Public domain dedication).
