# Supplementary material for: HALO: Study protocol for a single-case experimental design study evaluating the moderating impact of a befriending intervention on the association between loneliness and health in older adults
Source: HRB Open Res. 2022 Jan 31;3:60. Originally published 2020 Sep 4. [Version 2] doi: 10.12688/hrbopenres.13104.2 (PMC8825647; doi:10.12688/hrbopenres.13104.2)
Supplement: Peer review history

# Peer review: Reviewer response for version 2

**DOI:** 10.21956/hrbopenres.14723.r31373

**Published:** 2022-02-08

**Authors:** Frances Dark

**Affiliations:** 1 Metro South Addiction and Mental Health Services, Princess Alexandra Hospital, Woolloongabba, Queensland, Australia

I am happy to approve this amended article for indexing.

Is the study design appropriate for the research question?

Partly

Is the rationale for, and objectives of, the study clearly described?

Yes

Are sufficient details of the methods provided to allow replication by others?

No

Are the datasets clearly presented in a useable and accessible format?

Not applicable

Reviewer Expertise:

NA

I confirm that I have read this submission and believe that I have an appropriate level of expertise to confirm that it is of an acceptable scientific standard.

# Peer review: Reviewer response for version 1

**DOI:** 10.21956/hrbopenres.14207.r28860

**Published:** 2021-03-04

**Authors:** Frances Dark

**Affiliations:** 1 Metro South Addiction and Mental Health Services, Princess Alexandra Hospital, Woolloongabba, Queensland, Australia

This is an important area of research.

 The protocol needs brief additions:

- The use of the term "brain health" needs to be avoided or defined.
- The cognitive measures of "brain health" are not described in enough detail.
- Potential confounders based on the following factors need to be accounted for:
  - Personality style
  - Recent life events e.g. bereavement
  - Current living situation e.g. alone , partnered with family
  - Social contacts outside of alone
  - Other meaningful social or recreational activity etc.
- Also I would qualify the aim of the qualitative section- it could be interpreted as meaning you have ideas about potential mechanisms that you are going to explore ( qualitative design would not be a suitable method) or rather you will explore subjective understanding of benefit of befriending which may lead to testable potential mechanisms of impact of befriending on health ( if any).

Is the study design appropriate for the research question?

Partly

Is the rationale for, and objectives of, the study clearly described?

Yes

Are sufficient details of the methods provided to allow replication by others?

No

Are the datasets clearly presented in a useable and accessible format?

Not applicable

Reviewer Expertise:

Mental health, Health service research

I confirm that I have read this submission and believe that I have an appropriate level of expertise to confirm that it is of an acceptable scientific standard, however I have significant reservations, as outlined above.

# Response

**Published:** 2022-01-27

**Authors:** Joanna McHugh Power

**Affiliations:** National University of Ireland, Maynooth, Ireland

Dear Dr Dark,

 Thank you for your time and effort in reviewing our protocol. We have tried to respond carefully to each of your points, below, and made corresponding changes to our manuscript. (Reviewer comments in italics)

*The use of the term "brain health" needs to be avoided or defined.*

**Author Response**: The term “brain health” has now been defined in the introduction.

*The cognitive measures of "brain health" are not described in enough detail.*

**Author Response**: Thank you – we have provided more detail.

*Potential confounders based on the following factors need to be accounted for:*

-
  - *Personality style*
  - *Recent life events e.g. bereavement*
  - *Current living situation e.g. alone , partnered with family*
  - *Social contacts outside of alone*
  - *Other meaningful social or recreational activity etc.*

**Author Response**: Thank you for giving us these potential confounds to consider – we do not have the resources, currently, to include these factors in our design, particularly given the necessity to keep participant load as low as possible with the intensive longitudinal design, but would be interested in exploring in future.

*Also I would qualify the aim of the qualitative section- it could be interpreted as meaning you have ideas about potential mechanisms that you are going to explore ( qualitative design would not be a suitable method) or rather you will explore subjective understanding of benefit of befriending which may lead to testable potential mechanisms of impact of befriending on health ( if any).*

**Author Response**: We have used constructivist grounded theory to design and ultimately analyse our qualitative data. Constructivist grounded theory allows for, and encourages, the use of sensitising concepts (Charmaz, 2006, Gilgun, 2014), which, for us, are potential mechanisms through which befriending may impact health. We have explained this in our manuscript.  

- **Charmaz, K. **(2006). Constructing grounded theory: A practical guide through qualitative research. *SagePublications Ltd, London*.
- **Gilgun, M. **(2014). Deductive qualitative analysis and grounded theory: sensitizing concepts and hypothesis testing. . In *The SAGE Handbook of Current Developments in Grounded Theory* (ed. A. Bryant and K. Charmaz). Sage Publications: SI.

# Peer review: Reviewer response for version 1

**DOI:** 10.21956/hrbopenres.14207.r28207

**Published:** 2020-11-25

**Authors:** Felix Naughton

**Affiliations:** 1 School of Health Sciences, University of East Anglia, Norwich, UK

This is an excellent use of an aggregated within-person experimental design. The manuscript was very well written and clear. Some clarifications need to be made regarding power, but other than that this looks to be a high quality study.

 I am not so sure these are technically single cases experiments, as they are analysed together rather than N-of-1? The N-of-1 reference half-way through doesn’t help resolve this ambiguity. But this is very minor point.

 I wasn’t familiar with the ‘mezzo’ label before, and usually know this as the ‘meso’ level.

 The authors provide a very detailed description of the sample size estimation. It would be useful for them to add in the parameter estimates for hypothesis 2, as I was interested to read that the power would be greater for a moderation analysis than a main effect analysis (though this could be explained by my point in the paragraph below). Connected to this, brain health was listed as a key outcome measure in the hypotheses but did not feature in the power/sample estimation. Perhaps early on the EQ5D should be described as the primary outcome and ‘brain health’ as a secondary outcome?

 One potential flaw is that there will only be two observations before befriending occurs (in the ‘A’ period) with most observations happening in the ‘B’ intervention phase. I can see why this is done, from a pragmatic perspective, but this imbalance will reduce the power of the study compared to having equal measures between phases. This is the equivalent in a traditional experiment of having for example 110 people in the intervention arm and only 20 people in the control arm. The authors don’t seem to have considered this explicitly in their sample size/power estimation, so it’s not clear if they have taken this into account. The fact that the moderation analysis (testing interaction) has more power than the main effect analysis suggests perhaps this imbalance may have been taken into account? If not, this would make great a big difference to power.

 The authors suggest that if a participant discontinues using the befriending service, then they will move the participant (with their agreement) into an ABA N-of-1. While this is pragmatic, I am not sure how useful this data will be as this study isn’t set up as an N-of-1 as power is based on the aggregation of individuals. With only 13 data points per participant, and probably a limited number of intervention observations (during ‘B’) this may have very low power.

 Not being familiar with the ‘Delis Kaplan Executive Functioning System’ I suggest for readers like myself some explanation is provided to how it measures executive functioning.

 Should the authors consider in the discussion that being involved in a study and getting a telephone call every two weeks might itself reduce loneliness?

Is the study design appropriate for the research question?

Yes

Is the rationale for, and objectives of, the study clearly described?

Yes

Are sufficient details of the methods provided to allow replication by others?

Partly

Are the datasets clearly presented in a useable and accessible format?

Not applicable

Reviewer Expertise:

Health psychologist with experience of single case and within-person methodology.

I confirm that I have read this submission and believe that I have an appropriate level of expertise to confirm that it is of an acceptable scientific standard.

# Response

**Published:** 2022-01-27

**Authors:** Joanna McHugh Power

**Affiliations:** National University of Ireland, Maynooth, Ireland

Dear Dr Naughton,

 Thank you for your time and effort in reviewing our protocol. We note with thanks that you have approved the protocol’s publication. We also wish to respond to your thoughtful comments, in turn, below (Reviewer comments in italics).

 I *am not so sure these are technically single cases experiments, as they are analysed together rather than N-of-1? The N-of-1 reference half-way through doesn’t help resolve this ambiguity. But this is very minor point.*

**Author Response**: Thank you, we were not too sure about this either. We follow Hedges’ description of aggregated SCED, as cited. However, we have now removed the mention of N-of-1 in this article, since we agree it confuses things.

*I wasn’t familiar with the ‘mezzo’ label before, and usually know this as the ‘meso’ level.*

**Author Response**: It is ‘mezzo’ in the cited Berkman paper and causal cascade model, so we thought it would be most consistent to leave it as such!  

*The authors provide a very detailed description of the sample size estimation. It would be useful for them to add in the parameter estimates for hypothesis 2, as I was interested to read that the power would be greater for a moderation analysis than a main effect analysis (though this could be explained by my point in the paragraph below). Connected to this, brain health was listed as a key outcome measure in the hypotheses but did not feature in the power/sample estimation. Perhaps early on the EQ5D should be described as the primary outcome and ‘brain health’ as a secondary outcome?*

**Author Response**: Thank you, on reflection, we can see the confusion in the manuscript and have more clearly and consistently identified EQ5D as the primary outcome and brain health as secondary, throughout. With regards to the parameter estimates for hypothesis 2: the (higher) power of 0.9 in the manuscript is the power of the secondary outcome analysis (i.e. brain health) rather than the power of the moderation analysis, which we did not calculate.

*One potential flaw is that there will only be two observations before befriending occurs (in the ‘A’ period) with most observations happening in the ‘B’ intervention phase. I can see why this is done, from a pragmatic perspective, but this imbalance will reduce the power of the study compared to having equal measures between phases. This is the equivalent in a traditional experiment of having for example 110 people in the intervention arm and only 20 people in the control arm. The authors don’t seem to have considered this explicitly in their sample size/power estimation, so it’s not clear if they have taken this into account. The fact that the moderation analysis (testing interaction) has more power than the main effect analysis suggests perhaps this imbalance may have been taken into account? If not, this would make great a big difference to power.*

**Author Response**: The sample size estimation we used was based on the work of Shadish and colleagues (2014) and calculates a d statistic which takes into account, for AB ^k^ designs, number of time points per phase. In doing so, the imbalance between the A and the B period is taken into account. We agree that the pressure to conduct the trial in a pragmatic manner (i.e. having participants continue to receive the service they sought, rather than having them return to an “A” control period) does somewhat have the potential to adversely affect the power of the analysis – this is, we hope, offset by the use of this sample size estimator. We have edited the protocol manuscript to include a brief line providing more information about the sample size estimation (under “Sample”).

*The authors suggest that if a participant discontinues using the befriending service, then they will move the participant (with their agreement) into an ABA N-of-1. While this is pragmatic, I am not sure how useful this data will be as this study isn’t set up as an N-of-1 as power is based on the aggregation of individuals. With only 13 data points per participant, and probably a limited number of intervention observations (during ‘B’) this may have very low power.*

**Author Response**: Our aim was to analyse these cases on an individual basis rather than aggregated, as they arise. We agree this is likely to be underpowered but probably best seen as an exploratory supplemental analysis, designed to allow us to inspect trends in outcomes in ABA phases. We have clarified this in the manuscript.

*Not being familiar with the ‘Delis Kaplan Executive Functioning System’ I suggest for readers like myself some explanation is provided to how it measures executive functioning.*

**Author Response**: We now include a brief explanation of the Delis Kaplan system in the text, apologies for the omission.

*Should the authors consider in the discussion that being involved in a study and getting a telephone call every two weeks might itself reduce loneliness?*

**Author Response**: This is a concern in any study of loneliness – we have mentioned this in the discussion now.
